# Recent Findings on Exercise Therapy for Blood Glucose Management in Patients with Gestational Diabetes

**DOI:** 10.3390/jcm13175004

**Published:** 2024-08-23

**Authors:** Ah Reum Jung, Yongsuk Seo, Jooyoung Lee, Jae Gu Hwang, Somi Yun, Dae Taek Lee

**Affiliations:** Exercise Physiology Laboratory, Kookmin University, Seoul 02707, Republic of Korea; arjung@kookmin.ac.kr (A.R.J.); yseokss@kookmin.ac.kr (Y.S.); jylee@kookmin.ac.kr (J.L.); jaegu@kookmin.ac.kr (J.G.H.)

**Keywords:** pregnant women, gestational diabetes mellitus, type 2 diabetes mellitus, exercise guidelines, glucose management

## Abstract

Inadequate management of blood glucose levels in gestational diabetes mellitus (GDM) poses risks for both pregnant women and the developing fetus. Attaining appropriate blood glucose control is crucial to mitigate potential adverse outcomes. This study aimed to consolidate the latest guidelines from representative professional societies, providing insights into exercise therapy for GDM patients and suggesting potential avenues for future research. The review was conducted with up-to-date exercise guidelines from prominent societies, such as the American College of Obstetricians and Gynecologists (ACOG), the Society of Obstetricians and Gynecologists of Canada (SOGC), the Canadian Society for Exercise Physiology (CSEP), the American College of Sports Medicine, the American Diabetes Association (ADA), and the Korean Diabetes Association. The ACOG and SOGC/CSEP recommend 150 min of low to moderate intensity exercise, 3–4 times a week, combining aerobic and resistance exercises. All guidelines advise against activities involving sudden directional changes, physical contact, a risk of falling, and exercises performed lying down. Despite cautions from the ADA and ACOG on blood glucose fluctuations during physical activity, the lack of specific methods and recommendations from other societies reveals a notable gap in evidence-based guidelines for GDM. For effective and safe blood glucose management in GDM patients, further research should be conducted on the exercise-related precautions outlined for GDM patients. Establishing ample evidence would facilitate the development of customized exercise guidelines for GDM patients.

## 1. Introduction

A review indicated a consistent increase in the incidence of gestational diabetes mellitus (GDM) from 2011 to 2019, irrespective of race and ethnicity [1]. GDM is defined as diabetes diagnosed for the first time between the 24th and 28th weeks of pregnancy through an oral glucose tolerance test [2]. Similar to type 2 diabetes mellitus (T2DM), GDM primarily involves increased insulin resistance [3,4], but it possesses the characteristic of returning to normal glucose tolerance after delivery [5,6].

When adequate blood glucose management is not achieved during pregnancy, GDM poses risks for pregnant women, including preterm birth, dystocia, hydramnios, cesarean section, and heightened morbidity for type 2 diabetes post-childbirth [7,8,9,10]. However, GDM is not solely defined by high blood glucose levels; it also includes altered lipid status, oxidative stress, and low-level inflammation. These factors may persist even with well-managed glucose levels and can influence the risk of complications. Exercise may positively impact all these aspects, not just blood glucose management [11].

Complications for the fetus may involve shoulder dystocia, intrauterine death, neonatal hypoglycemia, macrosomia, and an increased risk of future obesity and diabetes [7,12,13,14]. Therefore, effective blood glucose management is crucial for GDM patients to prevent various complications that may arise for both the mother and the fetus.

The American Diabetes Association (ADA) recommends combining medical nutrition therapy with exercise therapy for effective blood glucose management in GDM patients [7]. Most people with GDM can manage fasting and postprandial hyperglycemia during pregnancy through dietary and lifestyle changes. However, about 15–30% of individuals with GDM require medication in addition to these modifications to achieve target blood glucose levels [15].

Exercise positively influences postprandial blood glucose, fasting blood glucose, and insulin dosage reduction. It also provides additional benefits, such as alleviating back pain, preventing excessive weight gain, reducing the frequency of cesarean sections, and enhancing psychological well-being [16,17,18,19,20,21,22]. However, large-scale exercise intervention studies specifically targeting GDM patients for blood glucose management are still limited [7]. Consequently, clear exercise guidelines for GDM patients during pregnancy for effective blood glucose management have not been conclusively established.

Therefore, the aim for this paper was to analyze the strengths and limitations of guidelines from prominent societies for pregnant women, including the American College of Obstetricians and Gynecologists (ACOG), the Society of Obstetricians and Gynecologists of Canada (SOGC), the Canadian Society for Exercise Physiology (CSEP), the American College of Sports Medicine (ACSM), ADA, and the Korean Diabetes Association (KDA). Additionally, this study was aimed to consolidate the key recommendations from these guidelines, providing the latest information on exercise therapy for GDM patients, and outlining future research directions.

## 2. GDM and Exercise Therapy

Exercise is highly beneficial for all diabetes patients and is an essential health management behavior [7]. However, the impact of exercise on blood glucose regulation may vary across different types of diabetes due to differences in pathophysiology and treatment options [23,24]. GDM, a unique condition during pregnancy, requires more careful management, as improper blood glucose control poses risks of complications for both the mother and the baby. GDM patients exhibit physiological differences in blood glucose regulation compared to healthy pregnant women, and those receiving insulin therapy have unique physiological characteristics related to exercise [20]. Therefore, specific recommendations and precautions for exercise need to be considered.

Unlike the exercise guidelines for type 1 diabetes mellitus (T1DM) and T2DM patients, which offer detailed recommendations for preventing exercise-related hypoglycemia/hyperglycemia, exercise-related blood glucose monitoring, and insulin dosage adjustments, there is a deficiency of specialized exercise guidelines for blood glucose management in GDM patients. Presently, exercise guidelines for healthy pregnant women are being adhered to without specific considerations for the distinctive characteristics of GDM [24,25]. Table 1 presents a comparison of the current exercise guidelines according to pregnancy status and diabetes types.

## 3. Pre-Exercise Assessment for Pregnant Women

### 3.1. Prohibitions Related to Physical Activity

Due to the unique condition of pregnancy, a pre-exercise assessment is essential for pregnant women before engaging in physical activity. Before participating in physical activity, pregnant women should evaluate potential obstetric or medical complications and any prohibitions before participating in physical activity. This is crucial because exercising with medical complications may not be safe for either the mother or the fetus. Also, consultation with an obstetrician is necessary to discuss the feasibility and adjustment methods for exercise therapy [26]. If exercise therapy is contraindicated, focusing on medication and nutritional therapy may be more advisable. The ACOG in 2015 [27] and the SOGC/CSEP in 2018 [28] outlined both absolute and relative contraindications for participation in physical activity (Table 2). There are some discrepancies in the conditions presented as contraindications by the two organizations [27,28]. As of 2021, the ACOG website notes the discontinuation of the 2015 clinical guidelines, recommending consultation of the 2020 clinical guidelines. In contrast to the 2015 version, the 2020 ACOG guidelines do not offer detailed information on factors considered as contraindications [29].

### 3.2. Pre-Exercise Assessment

Both the ACOG and SOGC/CSEP recommend regular physical activity for pregnant women if there are no obstetric or complicating contraindications. Additionally, they encourage pregnant women who were not engaged in regular physical activity before pregnancy to start exercising for the associated benefits [28,29].

The ACSM’s exercise guidelines suggest that pregnant women fill out a questionnaire and consult with an obstetrician to confirm their eligibility for exercise therapy. The ACSM recommends using the SOGC’s ‘Physical Activity Readiness Medical Examination for Pregnancy (PARmed-X for Pregnancy)’ to assess the suitability of regular exercise participation [30].

However, there has been criticism that existing questionnaires may emphasize prohibitions rather than permissions, potentially creating barriers to participation in physical activity. In response, in April 2021, the SOGC introduced the ‘Get Active Questionnaire for Pregnancy (GAQ-P)’ as a new screening tool to replace ‘PARmed-X for Pregnancy’. The GAQ-P consists of items to assess exercise-related contraindications and current physical activity levels to determine the applicability of exercise therapy. If there are changes in health status, the questionnaire should be filled out again [31].

## 4. Current Pregnancy Exercise Guidelines

While there is ample evidence supporting the recommendation of exercise therapy for managing T2DM, there is limited academic evidence regarding the impact of exercise on GDM management [32]. Currently, no specific exercise guidelines for GDM have been published, and the exercise guidelines for healthy pregnant women without complications are recommended for gestational diabetes patients [33]. The current exercise guidelines for pregnant women and GDM patients by the relevant societies are outlined in Table 3.

## 5. Comprehensive Recommendations Based on Exercise Guidelines

All of the academic institutions presented in Table 3 consistently propose exercise guidelines for pregnant women, suggesting ‘moderate-intensity aerobic exercise and resistance exercise for 150 min per week’ as a standard. Detailed information on common exercise guidelines for exercise volume (frequency, duration, intensity), exercise types, and criteria for exercise cessation for pregnant women is as follows.

### 5.1. Exercise Volume

The ACOG and SOGC/CSEP specify the recommended exercise volume in terms of exercise frequency, duration, and intensity. They advise a minimum of 3–4 days per week or daily exercise, with each session lasting 30–60 min, accumulating to over 150 min per week [28,29].

### 5.2. Exercise Intensity

Exercise intensity is generally categorized as moderate, with varying criteria for setting this level. Both the ACOG and SOGC/CSEP use heart rate, with the ACOG suggesting 60–80% of age-predicted maximum heart rate and the SOGC/CSEP recommending 40–59% of heart rate reserve as the moderate-intensity criteria [28,29]. The SOGC/CSEP further divides heart rate ranges based on age, recommending 125–146 beats/min for those up to 29 years and 121–141 beats/min for those 30 years and older [28]. The ACOG suggests that using the rating of perceived exertion (RPE) might be more effective, proposing an RPE of 13–14 (slightly hard) for pregnant women [29]. RPE is a method of expressing perceived exercise intensity on a scale of 6–20 and is widely used to gauge exercise intensity [36,37]. Another suggested method is the talk test, which assesses intensity by the ability to maintain a conversation during exercise [28,29,30].

### 5.3. Exercise Types (Recommended/Not Recommended)

#### 5.3.1. Recommended Exercises

All guidelines commonly recommend a combination of aerobic exercise and resistance exercise for pregnant women. The ACOG recommends walking, stationary cycling, aerobics, resistance exercise using body weight or elastic bands, stretching, and aquatic exercise, which are especially effective for pregnant women with back pain or immediately after childbirth [29]. The SOGC/CSEP emphasizes the combination of aerobic and resistance exercises and suggests that adding yoga or stretching may also be beneficial [28]. However, the ADA emphasizes that while flexibility exercises, like yoga or stretching, may be recommended for all types of diabetes patients, they cannot replace aerobic or resistance exercises for blood glucose control purposes [24].

#### 5.3.2. Not Recommended Exercises

Activities with a high risk of abdominal trauma or imbalance due to sudden directional changes, exercises involving physical contact, horseback riding, gymnastics, and other exercises with a risk of falling are generally not recommended for pregnant women, as stated in most guidelines [28,29]. Environmental factors should also be considered, and certain exercises, such as high-altitude activities and scuba diving, are not recommended during pregnancy due to environmental risks that can harm the fetus. These activities involve conditions where the fetus is vulnerable to decompression issues, such as changes in pressure and oxygen levels. The fetus is not equipped to handle the stress caused by these conditions, which could lead to serious complications [38,39]. Therefore, it is advised to avoid these activities to protect the fetus from potential harm.

### 5.4. Criteria for Exercise Cessation and Caution for Diabetes-Related Exercise

The ACOG and SOGC/CSEP provide criteria for stopping exercise during pregnancy. Symptoms, such as vaginal bleeding, regular uterine contractions, dizziness, shortness of breath, chest pain, abdominal pain, amniotic fluid leakage, calf pain, and swelling, are considered as reasons to cease exercise. The ACOG additionally recommends stopping exercise when experiencing headaches [28,29].

In the 2013 ADA guidelines, it is recommended that GDM patients using insulin should adjust food intake or insulin appropriately when participating in physical activity and that they should monitor blood glucose levels during exercise [24]. However, specific details regarding the necessary adjustments for food intake or insulin, as well as the recommended target range for blood glucose levels during exercise, are not provided. In 2020, the ACOG emphasized that high-intensity or prolonged exercise exceeding 45 min can induce hypoglycemia. In 2018, the ACOG also suggested that activities, such as walking for 10–15 min after each meal, could be generally beneficial for improving blood glucose control [29,40]. Other than these organizations, none of the guidelines from relevant societies provide specific recommendations on exercise precautions related to insulin administration for GDM patients, precautions for exercise-induced hyperglycemia and hypoglycemia, or carbohydrate intake guidelines based on pre-exercise blood glucose levels. When examining exercise guidelines for GDM patients provided by diabetes-related societies such as ADA and KDA, it becomes apparent that these guidelines recommend ACOG’s general exercise guidelines for healthy pregnant women rather than offering specialized content for GDM patients.

## 6. Analysis of Exercise Intervention Studies for GDM

Currently, exercise guidelines proposed by academic institutions for patients with GDM are based on the general guidelines for healthy pregnant women provided by ACOG [38]. While specialized exercise prescription guidelines for GDM patients have not been published by academic institutions, previous study suggested adhering to recommendations and precautions T2DM regarding hyperglycemia when GDM patients exercise [25]. Another previous study proposed that GDM patients receiving insulin treatment should follow the same recommendations as T1DM patients [41].

This analysis investigates whether there is any intervention research or academic evidence regarding exercise-related hypoglycemia prevention, precautions for exercise-related hyperglycemia, exercise-related blood glucose monitoring, and insulin dosage adjustment related to exercise for GDM patients.

In a previous study, eight GDM patients not receiving insulin reported that moderate-intensity walking 30 min after meals was beneficial for reducing postprandial blood glucose [42]. Another study involving 14 GDM patients revealed that starting moderate-intensity walking between 30 to 40 min after meals for about 20 min reduced peak blood glucose levels during the subsequent 2 h [43]. Thus, it was confirmed that performing moderate-intensity walking approximately 30 min after meals had a positive impact on postprandial blood glucose control in GDM patients.

However, many previous studies primarily focused on exercise intervention as a means to improve blood glucose control in GDM patients. To date, there has been no research on exercise intervention for GDM patients specifically addressing safe blood glucose ranges during exercise, snack consumption or insulin dosage adjustment based on pre-exercise blood glucose levels, precautions or considerations for hypoglycemia or hyperglycemia during exercise, etc. Moreover, even the few exercise intervention studies targeting GDM patients had extremely small sample sizes and limited research topics, usually 1–2 areas.

Furthermore, a caution was provided in a previous study [25], suggesting certain considerations, such as whether to initiate exercise based on blood glucose levels and recommending insulin adjustment before exercise to reduce the risk of hypoglycemia. However, this was based on study results targeting T2DM patients rather than being conducted on GDM patients. Another study [44] recommended preventing exercise-induced hypoglycemia in pregnant women with insulin treatment for gestational diabetes. The 2024 guidelines from the ADA recommend that patients with GDM engage in physical activity at least twice a week for a minimum of 20 to 50 min, focusing on moderate-intensity aerobic exercises, resistance training, or a combination of both [45]. These recommendations are based on evidence that physical activity can help manage blood glucose levels, reduce the need for insulin therapy, and lower insulin dosage in gestational diabetes patients. However, while these guidelines provide a minimum standard for exercise, a review of previous studies indicates that there is still a lack of specific exercise programs tailored to patients with gestational diabetes, as well as detailed guidance on precautions and considerations for this population [46]. A limitation of the study is that we are unable to report specific exercise guidelines for GDM, as these have not been sufficiently defined for this population.

## 7. Conclusions

Currently, there is a lack of research on specific exercise guidelines and cautionary requirements for effectively and safely improving blood glucose regulation in GDM patients. Consequently, reliable grades of recommendation and the strength of recommendations have not been established for exercise guidelines for GDM patients. As the effects of exercise can vary based on the type of diabetes and treatment methods, as well as pregnancy status, it is crucial to develop separate exercise guidelines for gestational diabetes based on its characteristics, rather than relying on guidelines designed for T1DM, T2DM, or healthy pregnant women.

Since exercise guidelines are clearly established for T1DM and T2DM, but not adequately defined for GDM, the following topics are proposed for future research in the field of exercise for GDM patients:

- Safe and optimal blood glucose levels for GDM patients during exercise.
- Optimal blood glucose levels and frequency of blood glucose monitoring for patients with GDM in relation to exercise.
- Potential risk factors and prevention methods for exercise-induced hypoglycemia in GDM patients receiving insulin.
- Guidelines for exercise-related insulin dosage adjustment in GDM patients.
- Guidelines for food intake based on blood glucose levels and exercise volume in GDM patients.
- Risk factors for exercise-related hypoglycemia and hyperglycemia in GDM patients not receiving insulin.

Research on this topic, supported by clear and reliable scientific evidence from pregnant women with GDM and incorporating rigorous validation, could enable the establishment of exercise guidelines. These guidelines would enhance safer and more effective blood glucose control and health management for patients with GDM.

## Figures and Tables

**Table 1:** Exercise guidelines for diabetes types and pregnancy conditions.

Classification	Pregnancy	Pregnancy and Diabetes	Non-Pregnancy and Diabetes	
Health complication	Healthy	GDM	T2DM	T1DM
Exercise recommendation	Recommended	Recommended	Recommended	Recommended
Purpose of exercise	Improving the health of pregnant women	Improving the health of pregnant women and achieving target blood glucose levels	Achieving target blood glucose levels	Preventing diabetic complications, improvement of cardiovascular fitness, and enhancement of physical fitness
Primary causes of blood glucose levels	-	Increased insulin resistance due to multiple placental hormones (human placental lactogen, estrogens, progesterone, cortisol, and placental growth hormone)	Increased insulin resistance	Insulin secretion deficiency
Medication	-	Insulin (only a minor percentage of GDM patients)	Oral hypoglycemic agent, insulin	Insulin
Effects of exercise on blood glucose regulation	Prevention of GDM onset	Blood glucose reduction during exercise	Blood glucose reduction during exercise	Minimal effect on blood glucose reduction
Application of exercise therapy	During pregnancy	From the 26th to 28th week of pregnancy until delivery	After being diagnosed with diabetes	After being diagnosed with diabetes
Existence of exercise guidelines	Yes	No specialized exercise guidelines for GDM	Yes	Yes
Organization for providing exercise guidelines	ACOG, SOGC/CSEP	ADA, KDA	ADA, KDA	ADA
Exercise guidelines	150 min per week of moderate-intensity aerobic exercise and resistance training	150 min per week of moderate-intensity aerobic exercise and resistance training	Moderate-intensity aerobic exercise and resistance training (150 min/week or high-intensity 75 min/week)	Moderate-intensity aerobic exercise, resistance training (150 min/week)
Preventive strategies for exercise-induced hypoglycemia	-	Not present	Caution when taking oral insulin-releasing agents Customized carbohydrate intake recommendations based on pre-exercise blood glucose levels	Determine carbohydrate intake recommendations based on pre-exercise blood glucose levels
Monitoring blood glucose in relation to exercise	-	Not present	Before and after exercise Changes in overall health, intensity of the exercise, or exercise duration Administration of insulin secretagogues or insulin Hypoglycemia or hyperglycemia	Before and after exercise Changes in overall health, intensity of the exercise, or exercise duration Administration of insulin secretagogues or insulin Hypoglycemia or hyperglycemia
Insulin dosage adjustment strategies related to exercise	-	Not present	Adjust meals and insulin based on exercise intensity and duration	Adjust meals and insulin based on exercise intensity and duration

ACOG, American College of Obstetricians and Gynecologists; SOGC/CSEP, Society of Obstetricians and Gynaecologists of Canada and the Canadian Society for Exercise Physiology; ADA, American Diabetes Association; KDA, Korean Diabetes Association; T1DM, type 1 diabetes mellitus; T2DM, type 2 diabetes mellitus; GDM, gestational diabetes mellitus.

**Table 2:** Absolute and relative contraindications for physical activity during pregnancy.

Absolute and Relative Contraindications to Exercise	ACOG (2015) [27]		SOGC/CSEP (2018) [28]	
	Absolute	Relative	Absolute	Relative
Ruptured membranes	O	-	O	
Premature labor	O	-	O	
Unexplained persistent vaginal bleeding	O	-	O	
Placenta previa	O	-	O	-
Preeclampsia	O	-	O	-
Incompetent cervix	O	-	O	-
Intrauterine growth restriction	-	O	O	-
High-order multiple pregnancy (e.g., triplets)	-	-	O	-
Uncontrolled type 1 diabetes	-	O	O	-
Uncontrolled hypertension	-	O	O	-
Gestational hypertension	O	-	-	O
Uncontrolled thyroid disease	-	O	O	-
Other serious cardiovascular, respiratory, or systemic disorder	O	-	O	-
Premature labor during the current pregnancy	O	-	-	-
Preeclampsia- or pregnancy-induced hypertension	O	-	-	-
Severe anemia	O	-	-	-
Recurrent pregnancy loss	-	-	-	O
A history of spontaneous preterm birth	-	-	-	O
Mild/moderate cardiovascular or respiratory disease	-	-	-	O
Malnutrition	-	-	-	O
Eating disorder	-	-	-	O
Twin pregnancy after the 28th week	-	-	-	O
Anemia	-	O	-	O
Unevaluated maternal cardiac arrhythmia	-	O	-	-
Chronic bronchitis	-	O	-	-
Extreme morbid obesity	-	O	-	-
Extreme underweight	-	O	-	-
History of extremely sedentary lifestyle	-	O	-	-
Orthopedic limitations	-	O	-	-
Poorly controlled seizure disorder	-	O	-	-
Heavy smoking	-	O	-	-
Other significant medical conditions	-	-	-	O

**Table 3:** Status of exercise guidelines by recognized organizations.

Classification		ACOG [29]	SOGC/CSEP [28]	ACSM [30]	ADA [16]	ADA [24]	KDA [23]
Target population		Women with uncomplicated pregnancies	All pregnant women without contraindication	Pregnant women	T1DM T2DM GDM	T1DM T2DM GDM	Women with diabetes
Components of exercise prescription	Exercise frequency	At least 3~4 days/week	Minimum 3 days/week	Most or all days of the week	Most or all days of the week	3~7 days	5~7 days
	Exercise Intensity	Less than 60~80% of age-predicted maximum maternal heart rate (usually not exceeding 140 beats/min) 13~14 (somewhat hard) on RPE Conversation during physical activity	Moderate intensity 40~59% HRR Conversation during physical activity	Conversation during physical activity	Moderate intensity	Moderate intensity 40~59% HRR 13~14 (somewhat hard) on RPE	Moderate or low intensity
	Exercise duration	30~60 min/day	At least 150 min/week	20~30 min/day	20~30 min/day	20~30 min/session	30 min/day At least 150 min/week
	Exercise recommended	Aerobic exercise: walking, stationary cycling, dancing, water aerobics Resistance exercise: using weights and/or elastic bands Stretching	Aerobic exercise: brisk walking, stationary cycling, swimming, aquafit Resistance exercise Stretching	Aerobic exercise: walking, swimming, stationary cycling, low-impact aerobics, running Resistance exercise Pelvic floor muscle training	Aerobic or resistance exercise	Aerobic exercise: walking, stationary cycling, aquatic activities Running (only if already highly active prior to pregnancy) Resistance exercise Prenatal yoga	Aerobic exercise
	Activities to be avoided	Activities at high risk of abdominal trauma or imbalance Scuba diving Exercise at altitudes above 1800 m Activities at excessive heats and humidities High intensity or prolonged exercise longer than 45 min	Activities at excessive heats and humidities Scuba diving Exercise at altitudes above 2500 m Activities at risk of falling or physical contact (horseback riding, downhill skiing, ice hockey, gymnastics)	Sports/activities that may cause loss of balance or trauma to the mother or fetus Activities at risk of falling, jumping, and quick changes in direction Scuba diving Hot yoga Exercise lying flat on the back after the 1st trimester Exercise using the Valsalva maneuver	Not present	Activities lying flat on the back after the 1st trimester Scuba diving Activities at risk of falling or physical contact (horseback riding, downhill skiing, water skiing, soccer, outdoor cycling, basketball, most racquet sports)	Not present
	Condition for discontinuation of exercise	Vaginal bleeding Amniotic fluid leakage Regular painful contractions Chest pain Abdominal pain Calf pain or swelling Dyspnea before exertion Dizziness Headache	Vaginal bleeding Amniotic fluid leakage Regular and painful uterine contraction Severe chest pain Increased shortness of breath during rest Dizziness or faintness during rest	Vaginal bleeding Amniotic fluid leakage Regular painful uterine contractions Chest pain Calf pain or swelling Increased shortness of breath Dizziness, syncope, or faintness during rest Headache	Not present	Vaginal bleeding Amniotic fluid leakage Regular painful uterine contractions Decreased fetal movement Dizziness Increased shortness of breath during rest Chest pain Calf pain or swelling Dizziness Headache	Not present
Considerations for exercise in diabetes	Safe blood glucose levels for GDM patients during exercise	Not present	Not present	Not present	Not present	Not present	Not present
	Medication	Not present	Not present	Not present	Understand risk of hypoglycemia during exercise for the 1st trimester	Not present	Not present
	Timing for exercise	Not present	Not present	Not present	Not present	Not present	Simple activities, such as walking for 10~15 min after each meal
	Exercise-related risk of hypoglycemia	High intensity or prolonged exercise longer than 45 min	Not present	Not present	Not present	Not present	Not present
	Adjusting medication for exercise	Not present	Not present	Not present	Not present	Appropriate food intake and insulin for insulin user during physical activities	Not present
Summary		Most detailed guidelines for pregnant women	Basic guidelines for pregnant women	Basic guidelines for pregnant women	Focus on type 1 and type 2 diabetes (T1DM, T2DM)	Guidelines centered More on pregnant women than GDM	Guidelines centered more on pregnant women than GDM
Referenced guidelines *		ACOG (2015) [27]	ACSM (2014) [34]	SOGC/CSEP (2018) [28] ACOG (2015) [27]	ACOG (2015) [27]	ACOG (2002) [35]	ADA (2021) [7]

ACOG, American College of Obstetricians and Gynecologists; ACSM, American College of Sports Medicine; SOGC/CSEP, Society of Obstetricians and Gynaecologists of Canada and the Canadian Society for Exercise Physiology; ADA, American Diabetes Association; KDA, Korean Diabetes Association; T1DM, type 1 diabetes mellitus; T2DM, type 2 diabetes mellitus; GDM, gestational diabetes mellitus; RPE, ratings of perceived exertion; HRR, heart rate reserve. * Reference guidelines used in the development of exercise guidelines.
